# The Role of *Culex pipiens* L. (Diptera: Culicidae) in Virus Transmission in Europe

**DOI:** 10.3390/ijerph15020389

**Published:** 2018-02-23

**Authors:** Victor A. Brugman, Luis M. Hernández-Triana, Jolyon M. Medlock, Anthony R. Fooks, Simon Carpenter, Nicholas Johnson

**Affiliations:** 1 Evolution Biotechnologies, Colworth Science Park, Sharnbrook, Bedford MK44 1LZ, UK; vab@evolutionbiotech.com; 2 Department of Disease Control, London School of Hygiene and Tropical Medicine, London WC1E 7HT, UK; 3 Animal and Plant Health Agency, Woodham Lane, Addlestone, Surrey KT15 3NB, UK; Luis.Hernandez-Triana@apha.gsi.gov.uk (L.M.H.-T.); tony.fooks@apha.gsi.gov.uk (A.R.F.); 4 Public Health England, Porton Down, Salisbury SP4 0JG, UK; jolyon.medlock@phe.gov.uk; 5 Health Protection Research Unit in Emerging Infections & Zoonoses, Porton Down, Salisbury SP4 0JG, UK; 6 Department of Clinical Infection, Microbiology and Immunology, University of Liverpool, Liverpool L69 7BE, UK; 7 The Pirbright Institute, Ash Road, Woking, Surrey GU24 0NF, UK; Simon.Carpenter@pirbright.ac.uk; 8 Faculty of Health and Medicine, University of Surrey, Guildford, Surrey GU2 7XH, UK

**Keywords:** *Culex pipiens*, West Nile virus, transmission, molestus, arbovirus, host preference

## Abstract

Over the past three decades, a range of mosquito-borne viruses that threaten public and veterinary health have emerged or re-emerged in Europe. Mosquito surveillance activities have highlighted the *Culex pipiens* species complex as being critical for the maintenance of a number of these viruses. This species complex contains morphologically similar forms that exhibit variation in phenotypes that can influence the probability of virus transmission. Critical amongst these is the choice of host on which to feed, with different forms showing different feeding preferences. This influences the ability of the mosquito to vector viruses and facilitate transmission of viruses to humans and domestic animals. Biases towards blood-feeding on avian or mammalian hosts have been demonstrated for different *Cx. pipiens* ecoforms and emerging evidence of hybrid populations across Europe adds another level of complexity to virus transmission. A range of molecular methods based on DNA have been developed to enable discrimination between morphologically indistinguishable forms, although this remains an active area of research. This review provides a comprehensive overview of developments in the understanding of the ecology, behaviour and genetics of *Cx. pipiens* in Europe, and how this influences arbovirus transmission.

## 1. Introduction

Mosquitoes are responsible for the biological transmission of a wide diversity of arboviruses (arthropod-borne viruses) that cause diseases in humans, companion animals and livestock [1]. Among the approximately 3500 mosquito species currently recognised worldwide [2,3], only a small number play a primary role in the transmission of arboviruses. The species that do fulfil this role tend to have adopted a degree of anthropophilic behaviour and occur at high abundance and in close proximity to susceptible hosts, primarily through exploitation of larval development sites created by humans.

In Europe, the recent emergence of mosquito-borne arboviruses has focused attention on identifying the species of mosquito that drive pathogen transmission. This phenomenon has occurred simultaneously with the incursion and establishment of several exotic and highly invasive species of mosquito associated with globalised trade. Several species of the *Aedes* genus have become established following importation and are a notable biting nuisance [4]. Significantly, they change the epidemiological status of the region with respect to the occurrence of vector-borne disease. The first incursion into Europe of *Aedes albopictus* (Skuse, 1895) was reported in Albania in 1979 [5], followed by Italy in 1990 [6]. The ability of this mosquito to exploit container habitats to breed in urban areas, produce diapausing eggs in temperate regions, and successfully expand through transportation in vehicles along highway systems, has facilitated its movement to more than 28 European countries and its establishment throughout large parts of the Mediterranean Basin [7], with a subsequent spread north. Similarly, since 2005 [8], populations of *Ae. aegypti* (Linnaeus, 1762) have been reported on the Portuguese island of Madeira and are expanding in the Black Sea region [9]; populations of *Ae. japonicus* (Theobald, 1901) have become widely established throughout Germany and have been reported from other countries such as Belgium and The Netherlands; and *Ae. koreicus* (Edwards, 1917) has been reported from Belgium and Italy [10,11]. The involvement of *Ae. albopictus* in the local transmission of arboviruses previously considered to be exotic, such as chikungunya virus (CHIKV) in Italy [12], is now a major concern for European public health. This species may furthermore facilitate emergence and re-emergence of other viruses including dengue virus (DENV).

In contrast, invasive species of mosquito appear to have had only a limited impact on the transmission of arboviruses that have a longer history of circulation in Europe. The highest profile of these is West Nile virus (WNV), which has been present in this region for at least twenty years. In southern Europe WNV has been detected in the indigenous mosquito species *Culex pipiens* (L. 1758) [13,14,15,16], which plays a primary role in transmission [17]. This observation has been supported by laboratory studies that demonstrated susceptibility to infection in *Cx. pipiens* and isolated virus in saliva from fully susceptible individuals. Transmission rates of between 37% and 47% have been reported for populations in Italy where the virus is endemic [18], compared to 33% for populations tested from The Netherlands [19], suggesting that WNV could emerge in northern Europe.

There is no evidence that WNV has reached mosquito populations north of countries surrounding the Mediterranean Sea or south-east Europe, despite the presence of *Cx. pipiens* in many of these areas. However, the related flavivirus, Usutu virus (USUV), was detected in southern Europe around the same time as WNV, and has emerged in northern European countries including Germany, The Netherlands and Belgium [20]. The principal vector for USUV is also *Cx. pipiens* and thus the reason for the absence of WNV in northern Europe is not fully understood, but among other factors may be related to the behaviour and distribution of different *Cx. pipiens* populations across Europe.

Previous reviews have considered the ecology of *Cx. pipiens* [21,22], current and future threats of mosquito-borne diseases across Europe [23,24] and the influence of a changing climate on vector-borne disease [25,26,27]. This review starts by presenting an overview of key arboviral threats to Europe, with focus on those for which *Cx. pipiens* is a vector. It then provides an updated overview of the literature relating to the taxonomy, ecology and behaviour of this important mosquito in Europe and examines future directions for research in these areas.

### 1.1. Viruses Associated with Transmission by Culex pipiens

At least ten arboviruses of medical and veterinary importance that are thought to be primarily transmitted by mosquitoes are currently circulating in Europe (Table 1). *Culex pipiens* has been shown to play a critical role in the transmission of three of these viruses. Conversely, there is no evidence that *Cx. pipiens* has contributed to the transmission of viruses such as DENV and CHIKV. Furthermore, experimental evidence overwhelmingly indicates that *Cx. pipiens* is refractory to Zika virus transmission [28,29,30,31,32,33,34,35,36], although some results are conflicting [37,38].

#### 1.1.1. Emergence of West Nile Virus in Europe

West Nile virus causes a febrile illness in both humans and horses that generally resolves without complications [44,45]. In a small proportion of cases (usually <1%), infected individuals develop more serious clinical symptoms and signs including encephalitis, meningitis and paralysis, followed by death in severe cases. The first isolation of WNV from the West Nile district of Uganda by Smithburn and co-workers is well documented [46]. Serum prepared from a blood sample taken from a febrile individual was inoculated into mice from which virus was isolated. Following its discovery, research in the 1950s in Africa identified an enzootic transmission cycle involving multiple bird species as natural reservoirs for the virus and mosquitoes as the primary vector group [47].

The majority of countries in sub-Saharan Africa have reported evidence of WNV presence, either through isolation of the virus or through seroprevalence studies [48]. North African countries including Egypt [49], Morocco [50] and Algeria [51] have also reported evidence of WNV. Due to its association with birds, avian migratory behaviour has been considered the most likely means for the translocation of WNV from Africa to northern latitudes [52]. Repeated emergence of WNV in Israel, Italy, France and Spain correspond to bottlenecks of major flyways of migratory birds travelling north as they avoid the Mediterranean Sea, or cross at its narrowest points [53].

West Nile virus has caused sporadic outbreaks of disease in southern Europe in both humans and horses [54]. Phylogenetic analysis has demonstrated at least eight distinct lineages of WNV, of which two, lineages 1 and 2, circulate in Europe [55]. The most significant epidemic occurred in south eastern Romania with a focus in the capital, Bucharest. Almost 400 cases of encephalitis and 17 deaths were reported in 1996, with further cases reported in subsequent years [56]. The Danube delta was considered the potential site of introduction but with transmission in an urban setting. Interest in WNV was also stimulated by its emergence in North America in 1999, initiating a major epidemic outbreak and highlighting its epidemic potential in other countries [57]. The virus caused numerous cases of disease in birds, particularly North American crows (*Corvus brachyrhynchos*), domestic horses and humans. A wide range of indigenous mosquito species were found to be infected with WNV [58], however, the *Cx. pipiens* complex was considered to be the principal vector [59,60].

During the first decade of the 21st century, there was an increase in the number of detections of WNV outbreaks in Europe. It remains unclear if this phenomenon was due to more frequent annual re-introduction of WNV or a greater focus on surveillance in the Mediterranean Basin. The successful overwintering of virus in mosquito populations in Italy between 2008 and 2011 was a notable epidemiological shift [61]. The virus causing the outbreaks in Italy and in other countries was identified as belonging to WNV lineage 1 [62]. West Nile virus lineage 2 was first detected in Hungary during 2004 and then spread west and south, reaching Greece prior to 2010. The outbreak in Greece was notable for involving a high number of human cases, including 33 deaths attributed to the infection [14]. Mosquito species from the genus *Culex*, *Aedes* and other mosquito genera have been shown to transmit WNV under experimental conditions but the epidemiological significance for natural virus transmission is unclear [63].

#### 1.1.2. Emergence of Usutu Virus in Europe

Usutu virus was first detected in Europe in dead blackbirds (*Turdus merula*) collected following a wild bird die-off event in Tuscany, Italy, in 1996 [64]. A later emergence in 2001 is better documented due to a highly visible die-off of birds around Vienna, Austria. Submission of blackbirds, great gray owls (*Strix nebulosa*) and a barn swallow (*Hirundo rustica*) resulted in detection of virus by histopathology and reverse transcription polymerase chain reaction (RT-PCR) [43]. Usutu virus has emerged in countries across southern Europe and it has subsequently spread north across western and central Europe [65,66]. A small but growing number of documented cases of human infection with USUV have been recorded, although these have often been in patients with additional underlying health conditions [67,68]. However, most cases of USUV infection appear to be asymptomatic [69]. 

Phylogenetic analysis using complete USUV genomes suggests that there have been multiple introductions of the virus into Europe over the past 50 years and that migrating birds are the most likely mechanism of translocation over long and short distances [20]. *Culex pipiens* originating from a colony established in the Netherlands have been shown experimentally to be highly susceptible to infection with USUV when compared to WNV, although the ecoform status of the mosquitoes used was not explored [19].

#### 1.1.3. Sindbis Virus in Europe

Sindbis virus (SINV) was first isolated from a pool of *Cx. pipiens* and/or *Culex univittatus* (Theobald, 1901) mosquitoes collected from the Sindbis health district, 30 km north of Cairo, Egypt [70]. Infection causes a rash and long-lasting polyarthritis that has been recognised in northern Europe for decades [23,71]. It is known colloquially as Ockelbo disease in Sweden, Pogosta disease in Finland and Karellian fever in Russia. In South Africa it has been reported to cause disease in horses [72]; SINV infection in horses or other domestic animals has not been observed in Europe, possibly due to a lack of surveillance. The virus circulates between birds and mosquitoes with occasional spill over into human populations [73]. Phylogenetic analysis of SINV suggests that there is long distance translocation of the virus, possibly through bird migration [74].

Experimental studies have shown that a range of mosquito species present in Scandinavia are capable of transmitting SINV but that *Culex torrentium* (Martini, 1925) demonstrated higher infection and transmission rates than *Cx. pipiens* [75,76]. Subsequent field studies have shown higher rates of SINV infection in wild caught *Cx. torrentium* than in other species [77] and this is now considered the most important vector species. Although *Cx. torrentium* is found across Europe and the Middle East, few cases of SINV are reported outside of northern Europe, and are limited to occasional virus isolations [78]. The susceptibility to infection of *Cx. torrentium* for WNV or USUV has not been defined [79].

#### 1.1.4. Other Viruses Transmitted by *Culex* Mosquitoes

The other *Culex*-transmitted viruses detected in Europe include Lednice virus (LEDV) and Rabensberg virus (RABV). LEDV, a bunyavirus, was isolated from *Culex modestus* (Ficalbi, 1889) in the Czech Republic in 1963 [80]; this mosquito remains the only known vector [81]. RABV is a more recent isolation from the Czech Republic and is a virus related to WNV. It was first isolated from pools of *Cx. pipiens* collected in 1997 from South Moravia near the border with Austria [82]. Batai virus (BATV) was originally detected in *Culex gelidus* (Theobald, 1901) in Malaysia in 1955 [24], but in Europe it has been associated with Anopheline species [83].

### 1.2. Culex pipiens Taxonomy

The taxonomy of the *Cx. pipiens* complex remains a much debated subject due to the morphological similarity between some species and the varied behaviours exhibited within species [84,85,86,87]. The first description of *Cx. pipiens* is attributed to Carl Linnaeus in 1758. The complex (or assemblage [86]) of species includes *Cx. pipiens*, *Cx. quinquefasciatus* (Say, 1823), *Cx. australicus* (Dobrotworsky and Drummond, 1953) and *Cx. globocoxitus* (Dobrotworsky, 1953) with varied geographical distribution that has been modified by the translocation of species between continents [88]. Additionally, some authors include the sibling species *Cx. torrentium* in taxonomic studies of the complex owing to its similar morphology and larval ecology [85,89]. Within the species *Cx. pipiens* there are two ecoforms (sometimes called biotypes) recognised, pipiens (L.) and molestus (Forskål, 1775), based primarily on ecological and behavioural traits. The term molestus was first introduced by Petrus Forskål who recognised the species during an expedition to Egypt and the Arabian Peninsula. The behavioural and physiological traits reported as broadly separating the two forms are summarised in Table 2.

Evidence from several studies of European *Cx. pipiens* populations has indicated that ecoform molestus is a distinct species separate from ecoform pipiens and arose from a single speciation event [85,90,91]. This contrasts with the alternative theory that molestus populations arose from repeated and independent colonisations of underground habitats by aboveground pipiens populations [92,93,94]; other studies have shown equivocal results [95].

Herein, we use the following terms: (1) “*Cx. pipiens* complex” when referring to the group as a whole, (2) “*Cx. pipiens*” when referring to specimens separated from *Cx. torrentium* but no further, (3) “pipiens” and “molestus” in reference to the ecoforms, and (4) “pipiens/molestus” and “pipiens/quinquefasciatus” in reference to hybrid forms where appropriate.

### 1.3. Delineation of Species, Ecoforms and Hybrids

Differences in the structure of the male genitalia can be used to distinguish members of the complex [85]. However, the lack of distinguishing morphological features to separate females adds complication to the identification of surveillance trap catches where females are usually the target. The presence or absence of behavioural traits such as autogeny (Table 2) have been used to identify between the forms; however, this approach is not a consistently reliable method for separating the ecoforms. Furthermore, demonstrating autogeny in wild-caught populations is labour intensive, requiring the collection and rearing of larvae, and is therefore impractical for large scale screening. This has led to the development of several molecular techniques for differentiating the two ecoforms and their hybrids (Table 3).

**Figure 1:**
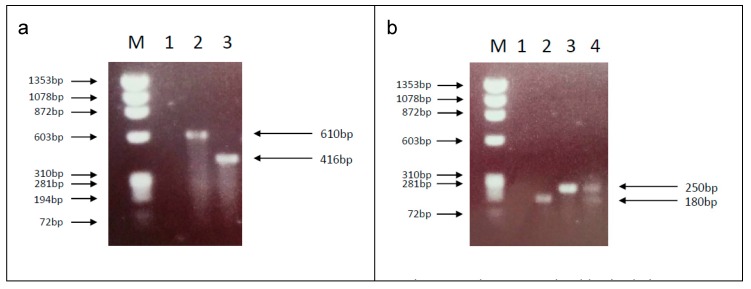
Gel images showing discrimination between (**a**) *Culex torrentium*/*Culex pipiens* [M = ϕX174 marker, 1 = negative control, 2 = *Cx. pipiens*, 3 = *Cx. torrentium*] and (**b**) *Cx. pipiens* form pipiens, *Cx. pipiens* form molestus and hybrid forms [M = ϕX174 marker, 1 = negative control, 2 = form pipiens, 3 = form molestus, 4 = pipiens/molestus hybrid].

Initial differentiation techniques were aimed at identifying polymorphisms at 20 loci in order to differentiate above and belowground breeding populations associated with the London Underground, and to examine gene flow [92]. This method was developed to include sequence comparison of up to 11 concatenated sequences to enable phylogenetic distinction of the two ecoforms [85]. An alternative approach compared polymorphic microsatellite markers amplified to generate fingerprints for autogenous and anautogenous populations [90,96]. Subsequent methodologies have largely been based on the polymerase chain reaction (PCR), DNA sequencing or restriction fragment length polymorphism (RFLP) (Table 3). Many of these have focused on a single locus to distinguish between the two forms, particularly the CQ11 locus [97] (Figure 1). This end-point PCR approach is often preceded by the use of a multiplex PCR to separate *Cx. torrentium* from *Cx. pipiens* [98] (Figure 1), although identification via comparative wing morphometrics can be used for this [99]. In a further modification, fluorescent probes have been developed that selectively bind to the polymorphisms within the same real-time PCR amplification [100]. Some authors have expressed caution in using only a single diagnostic marker for the identification of the *Cx. pipiens* complex [101,102], and advocate the use of multiple targets for maximum taxonomic clarity. For example, although a nucleotide substitution from G to A at the 3rd position of the 68th codon of the COI gene was reported as being diagnostic for form molestus over form pipiens [91], this finding was not replicated in a subsequent UK study that targeted the same region [103]. In an attempt to avoid differences between assays, a recent study employed a combined four-point approach to characterising Mediterranean *Cx. pipiens* populations, using assays targeting the CQ11, *ace-2*, COI and *Wolbachia* (*w*Pip) infection typing markers [102].

An alternative approach to species delineation is the application of matrix-assisted laser desorption/ionization time-of-flight mass spectrometry (MALDI-TOF MS). While this application is still in relative infancy, it has been used for the identification of various vector groups [111,112,113] and in future it may be possible to use this to define ecoforms of *Cx. pipiens* based on changes in protein expression.

### 1.4. Distribution and Hybridisation

*Culex pipiens* is widely distributed across Eurasia and further afield [22,90]. Our understanding of the local and regional distribution of its ecoforms has, however, developed only relatively recently, aided by the increasing use of molecular species delineation methods. There remains, however, a poor understanding of the relationship between the genetics of the ecoforms and their phenotype [103]. Initial evidence indicated a fairly consistent separation between the habitats of each ecoform: the ubiquitous pipiens ecoform was associated with natural and artificial aboveground habitats across rural and urban areas and the molestus form was found in urban underground habitats [22]. Particularly in northern Europe, this habitat distinction was believed to serve as a barrier to hybridisation between the forms and this was supported by limited success in breeding between forms under laboratory conditions [90,92].

**Figure 2:**
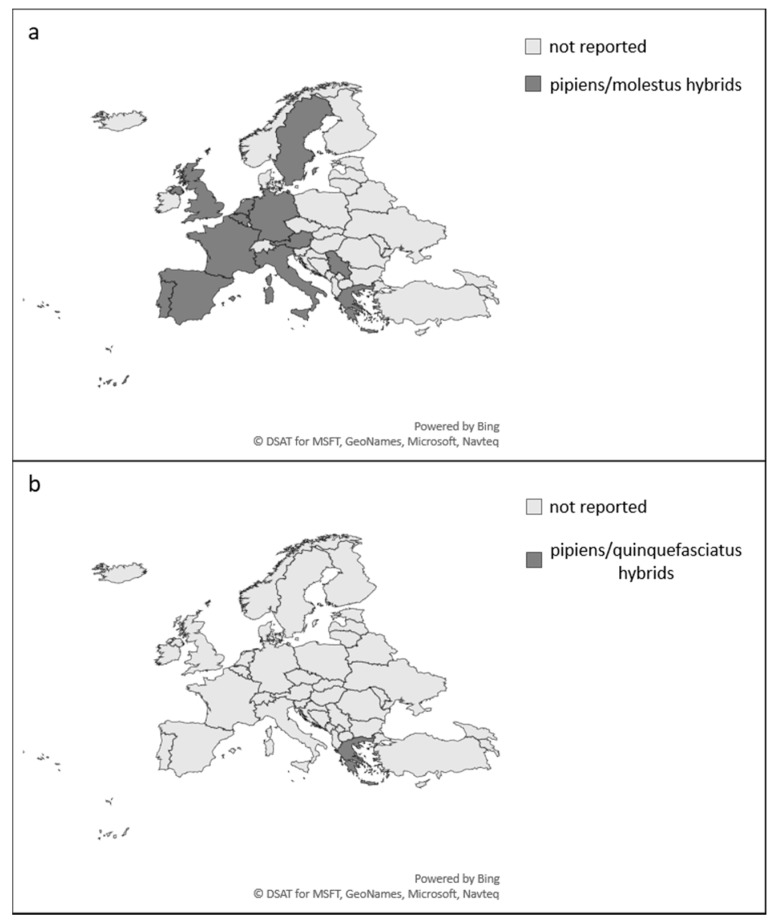
European country-level reports of natural hybrid populations of (**a**) *Culex pipiens* forms pipiens/molestus hybrids; (**b**) *Culex pipiens*/*Culex quinquefasciatus* hybrids. References available in Appendix A.

Present evidence, however, suggests that this habitat separation is far less rigid, with cross-breeding experiments and analysis of genetic markers from field and colony specimens indicating that inter-breeding populations of pipiens and molestus can be found sympatrically in both above- and belowground urban habitats, as well as in rural and semi-rural areas [100,103,110,114,115,116,117,118,119]. Indeed, natural hybrid pipiens/molestus forms have now been reported from at least 12 European countries (Figure 2a) with reported rates of hybridisation of up to 25.7% [116]. The relative abundance of each of the forms and hybridisation rates have been found to vary across latitudes, with the proportion of molestus populations relative to pipiens increasing from northern to southern latitudes [120]. To add further complexity, hybridisation of *Cx. pipiens* with *Cx. quinquefasciatus* has been reported from the Mediterranean Basin (Figure 2b) [102,121,122], despite sympatric populations of these species existing without hybridisation in East Africa [123].

The occurrence of natural hybrid populations has important consequences for the risks of pathogen transmission [124]. Changes to mosquito host preference, vector competence, the occurrence of autogeny and the ability to forgo diapause and continue reproduction through the winter months may all alter virus transmission dynamics. This may have contributed to the persistence of WNV in Romania during the 1990s where the presence of mosquitoes indoors and in flooded basements were considered risk factors for human infection [125]. Additionally, the strains of the endosymbiont *Wolbachia pipientis* associated with *Cx. quinquefasciatus* and the different ecoforms of *Cx. pipiens* differ [102], and the impact of such differences on vector competence is not fully understood. Studies of *Cx. pipiens* populations in Portugal demonstrated that gene flow occurred predominantly from the molestus to the pipiens form [119]. Asymmetric gene flow in this fashion could alter feeding preferences of *Cx. pipiens* from an ornithophilic to mammalophilic feeding preference, as demonstrated in the USA [126]. The vector competence of molestus populations to WNV in The Netherlands was lower (6–10%) than that of pipiens (0–32%) and hybrid (0–14%) forms [127]. In this context, gene flow from pipiens to molestus could result in increased vector competence and thus may be equally important in influencing local pathogen transmission dynamics.

**Figure 3:**
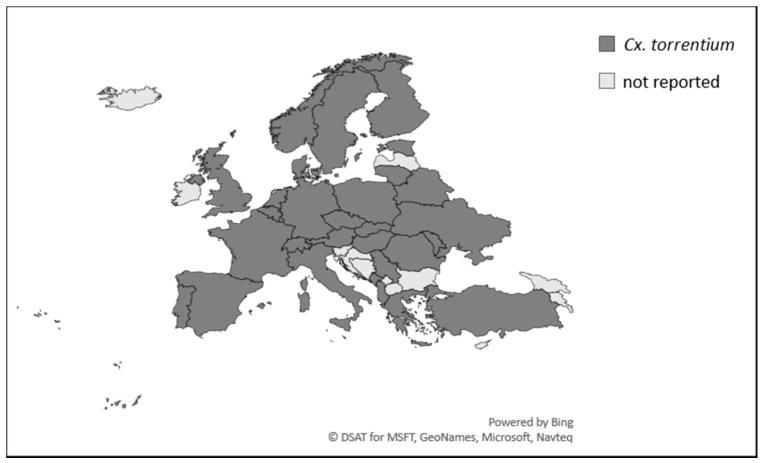
European country-level reports of *Culex torrentium*. References available in Appendix A.

*Culex torrentium* has also been reported from many countries across Europe (Figure 3) where its larvae are often found in sympatry with *Cx. pipiens* [89,114,128,129]. In many studies little morphological separation is performed [79], thus masking the true distribution of the two species. Initially believed to be a rare European species [130], *Cx. torrentium* is now recognised to be widespread in northern and central regions of Europe [79,89]. When compared with *Cx. pipiens*, these species form an apparent contrasting gradient of abundance: in northern regions *Cx. torrentium* dominates, in central Europe both species exist in similar proportions, and in southern Europe *Cx. pipiens* is the dominant species and *Cx. torrentium* is rarely reported [89]. The current distribution of *Cx. torrentium* may reflect a range expansion, perhaps in response to favourable anthroponotic environmental changes [110], but the misidentification of females as *Cx. pipiens* prior to the widespread use of molecular analyses may have hindered information on its distribution.

### 1.5. Culex pipiens Blood-Feeding Behaviour

A critical behavioural trait relevant to arthropod-borne virus transmission is a vector’s host feeding pattern. Host selection determines the exposure of a mosquito to pathogens and its involvement in enzootic, zoonotic or anthroponotic transmission cycles [131]. Host selection by mosquitoes is a complex phenomenon, influenced by an interplay of genetic and environmental factors [132]. The latter includes the local and seasonal presence of vertebrate hosts [133], host defensive behaviour against biting [134] and the presence of pathogens in the arthropod, host, or both, which may influence rates of vector-host contact [135,136,137,138,139]. Evidence for preferential feeding on specific hosts may be derived from studies that identify the blood meal hosts of wild-caught engorged mosquitoes, or semi-field or laboratory tests offering a choice of feeding from different hosts [132].

The pipiens ecoform is considered to be almost exclusively ornithophilic (bird-feeding), whilst the molestus ecoform feeds on other mammalian hosts, including humans [140,141] (Table 2). Here, we collated data from 29 European studies identifying the blood meals of *Cx. pipiens* (Table 4).

**Figure 4:**
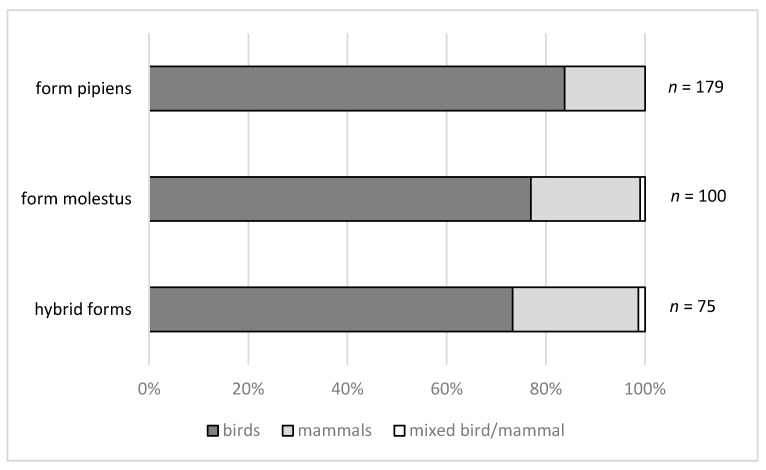
Proportion of blood meals of *Culex pipiens* taken from birds, mammals or mixed bird/mammal sources. Data collated from [117,147,149].

Collectively, these data show feeding of *Cx. pipiens* on a wide range of hosts encompassing mammals (eight orders, 12 families and 17 species), birds (14 orders, 33 families, 82 species) and reptiles (two orders, three families, three species). Eight of these studies identified specimens to ecoform, and three of these [117,147,149] successfully collected blood-fed specimens of both ecoforms and their hybrids, identified by sequence analysis of the CQ11 locus. Collectively, these latter three studies identified both ecoforms and their hybrids as feeding on both mammals and birds. Interestingly, all found that birds were highly utilised by the pipiens and molestus ecoforms plus their hybrids (Figure 4), with no significant differences in feeding preference between the forms. These results contrast with findings in the USA showing that specimens with a higher proportion of molestus ancestry fed more frequently on humans [170,171]. Reasons for these disparate findings may lie with geographic or seasonal differences in host availability, the relatively low sample sizes inherent with the challenges of collecting blood-fed specimens, or with differences in the microsatellite markers used to identify the forms in each study.

Relatively few manipulative comparisons of host selection, whereby mosquitoes are offered choices to feed on different hosts, have been carried out with *Cx. pipiens* under field, semi-field or laboratory conditions. Preferential attraction was recorded towards chicks by the pipiens ecoform, to humans by the molestus ecoform, and intermediate feeding behaviour in pipiens/molestus hybrids from field-collected populations in Chicago, USA [126]. Choice tests can be an effective method to compare feeding preferences between individual hosts, but to our knowledge, these have not been conducted to compare the ecoforms and hybrids of European populations of *Cx. pipiens*.

Field studies collecting mosquitoes attempting to feed on live human or animal baits can also greatly contribute to our understanding of host preference [172]. Several field studies have reported human-biting *Cx. pipiens*; studies in Portugal [149] and the UK [173] collected both pipiens and molestus ecoforms by human landing catch. Although the study in Portugal identified human blood in one engorged pipiens female [149], the specimens collected by human landing catch in both studies did not contain blood to permit confirmation of human feeding. However, this collection method is considered the gold-standard approach for assessing mosquito-human contact rates, with mosquito feeding (or at least probing) assumed to occur after landing [172]. Combining these field data with laboratory choice tests and, although challenging, with blood meal studies that are coupled with comprehensive surveys of vertebrate hosts in the sample area to assess the impact of host availability, will contribute further to our understanding of host selection and preference of members of the *Culex pipiens* complex. However, studies where wild mosquitoes are offered a choice of host are very rare and findings such as those reported above could therefore represent opportunistic feeding rather than a true preference.

## 2. Future Research Directions

Our understanding of the *Cx. pipiens* complex has expanded rapidly in recent years, but there remain many intriguing and as yet unexplored questions concerning their biology and ecology. Below we highlight four areas of research important to defining the impact of *Cx. pipiens* on present and future virus transmission in Europe.

(1) What factors lead to successful arbovirus transmission by populations of *Culex pipiens*?

The distribution of *Culex*-transmitted arboviruses is not uniform across Europe. Identification of the different factors that lead to successful transmission of viruses and those that preclude virus emergence are critical to understanding this distribution. Northern Europe has seasonally abundant populations of *Cx. pipiens* that appear to support transmission of USUV but not WNV [174]. This suggests environmental and climatic factors alone cannot explain the absence of WNV from countries such as Germany, Poland, The Netherlands and the United Kingdom. In North America, *Culex* species, including *Cx. pipiens*, enabled rapid spread of West Nile virus across the continent with no apparent barriers. Expanding upon recent work [120] investigating the distribution of the ecoforms of *Cx. pipiens* across Europe is essential to furthering our understanding of the relationship between the ecoforms and their hybrids with current arbovirus distribution patterns. Furthermore, as many important arboviruses exist in bird-mosquito-bird transmission cycles, identifying hotspots of high mosquito and resident and migratory bird populations will enable better targeting of interventions in advance of a novel virus introduction. Such hotspots may include rural wetland areas [168,175] but could, increasingly, include more urbanised areas [176,177,178]. At the level of the mosquito, there remain many questions regarding the complex interplay of genetic and environmental factors that influence vector competence and mosquito-virus-host interactions. These include the extrinsic incubation period, viral adaptivity, mosquito and host immunity and mosquito behaviour. In reference to the latter, newly-emerged Australian ecoform molestus females preferentially delay blood-feeding until after laying their first egg batch [179]. If such high levels of obligatory autogeny exist in European populations, this would not only provide a highly beneficial population survival mechanism but may influence the transovarial maintenance of virus through several generations. Finally, the survival of virus in overwintering *Cx. pipiens* is likely a critical factor involved in the maintenance of transmission cycles in Europe; a recent study detected WNV RNA in overwintering *Cx. pipiens* in the Czech Republic [180]. Further investigation of the factors influencing overwintering survival, post-hibernation emergence, and subsequent dispersal of *Cx. pipiens* and its ecoforms, as conducted elsewhere [181,182], will improve our understanding of the role of overwintering in virus maintenance, particularly in regions of Europe that experience colder winters.

(2) What are the potential impacts of a changing environment?

That climate changes are occurring and will impact both native and non-native arthropod fauna worldwide is well established. The potential influences on arthropod-borne pathogens have been explored [25,26,27], although the specific effects will vary considerably according to mosquito species biology and the region concerned [183]. Anthroponotic changes influencing the structure of the environment may be equally important in altering mosquito populations at the local or regional scale [184,185]. For example, the creation of urban wetlands as part of sewage treatment works [178] could increase available eutrophic habitat particularly suitable for ecoform molestus [85]. Increasing urbanisation could provide additional container habitats suitable for existing urban mosquito populations, or facilitate an adaptive shift by other species towards the utilisation of urban habitats, as evidenced by an increasing urban population of *Anopheles plumbeus* (Stephens, 1828) in various parts of north-western Europe [176,177,186,187]. Urban centres could be at further risk of vector-borne disease if existing temperature rises were compounded by the urban heat island effect in such locations, although the precise effects of this phenomenon on pathogen transmission risk are likely to be complex [185,188]. The storage of water during periods of drought could additionally provide increased urban habitat for mosquito breeding [189], whilst the reversion of arable land to wetlands could provide further habitat for *Culex* mosquitoes and provide a location where grazing animals come into contact with migratory birds [175].

(3) What are the key factors influencing rates of hybridisation?

The variable rates of hybridisation in European populations between sympatric populations of pipiens and molestus ecoforms indicate the existence of multiple barriers to hybridisation that extend beyond simple allopatric reproductive isolation. Although in parts of Europe hybridisation rates are low, rates in southern Europe may approach those reported from northern Africa [122,190]. To what extent reproductive barriers are behavioural, such as environmental requirements for swarm formation or specificity of matched wing beat frequencies [191,192], or intrinsic, for example mediated by commensal *Wolbachia* strains and cytoplasmic incompatibility [109,193], is currently unknown. Furthermore, although human-mediated transport of mosquitoes may facilitate long-distance species translocation and provide opportunities for hybridisation aboveground [121], to what extent are belowground molestus populations able to disperse within and beyond their existing habitats? Approaches such as the use of mark-release-recapture aided by fluorescently- or immune-marked insects [194,195] in belowground systems could, for example, reveal the dispersal potential of form molestus.

(4) How do the olfactory responses to semiochemicals of host and environmental origin differ?

Furthering the understanding of the responses of the *Cx. pipiens* complex to volatile compounds produced by vertebrate hosts, nectar sources and larval habitats will facilitate the development of novel repellents, attractants and more optimal approaches to surveillance and control. To date, the olfactory responses of *Culex* species to host odours have been investigated for *Cx. quinquefasciatus* [196,197], and to flower odours in ecoform pipiens [198] and molestus [199]. However, directly comparative studies of the olfactory responses between the ecoforms have not been conducted, and paired trap comparison studies comparing above- and belowground collections remain unexplored. Recent work has shown that ecoforms pipiens and molestus, plus their hybrids, were collected in similar ratios by BG-Sentinel and Mosquito Magnet Liberty Plus traps [120]. However, *Cx. torrentium* was found to be under-represented in CDC light trap catches in Germany and Sweden in comparison to *Cx. pipiens* [200,201] and although the authors did not molecularly identify specimens to ecoform, these results illustrate the need for further field investigation using other trap types.

In summary, it is vital that data on members of the *Cx. pipiens* complex is collected from countries across Europe and at a range of geographic scales that reflect different ecological zones. Comparisons should also be made between urban and rural populations and those in intermediate areas. Habitat differences may be more important in influencing distribution and hybridisation rates than broader latitudinal trends [118,120]. Studies conducted at the regional, national and pan-European level will provide critical data to model trends in mosquito biology and virus transmission, and to better inform regional approaches to surveillance and control. However, these large-scale studies cannot replace targeted field-based studies which are critical to understand the factors influencing transmission at the level of the vector and its hosts in different local habitats. Finally, although these research questions span several fields, it has become increasingly clear that future studies should, insofar as is possible, identify *Cx. pipiens* to the level of both species and ecoform. The continued decrease in costs and increase in the speed of molecular identification approaches will no doubt greatly contribute towards this goal.

## 3. Conclusions

Current evidence from across Europe highlights the importance of the *Cx. pipiens* complex in the current and potential future transmission of important medical and veterinary arboviruses. It is therefore imperative that a concerted effort be made between research and governmental agencies across Europe to better target future sampling efforts to answer the remaining questions concerning the ecology and genetics of mosquito and pathogen that influence this association.

Surveillance for mosquito-borne viruses in mosquito populations varies widely across Europe [17]. Extensive surveillance is conducted in northern Italy where cases of WNV occur annually in an attempt to detect virus in mosquitoes populations [202]. This offers the opportunity for public health authorities to warn health professionals before the occurrence of human disease. Both Germany and Switzerland conduct extensive surveillance to detect invasive mosquitoes and the emergence of virus infections. This has proven useful in mapping the spread of USUV across Europe. In the majority of countries across Europe, however, surveillance is reactive in response to disease outbreaks or changes in the mosquito population [203].

The extent of the distribution of the specific forms of *Cx. pipiens* is just beginning to be defined. However, evidence indicates that latitudinal differences in the distribution of *Cx. pipiens* forms and their hybrids, together with the distribution of the sibling species *Cx. torrentium*, may influence the transmission dynamics of arboviruses in Europe. However, the picture is more complicated than simply this fact and will include the effect of different environmental conditions on the life cycle and behaviour of the mosquitoes, as well as intrinsic factors such as vector competence. In addition, despite the importance of this species in current and potential pathogen transmission, increasing our understanding of how species complexes as a whole function within an ecosystem to contribute to pathogen transmission is vitally important. For example, Rift Valley fever virus outbreaks involve multiple species that act sequentially depending on environmental circumstances. Therefore, maintenance of surveillance approaches that target a wide range of mosquito species should be used.

Current evidence continues to support the importance of birds as a major blood-meal host for *Cx. pipiens* across Europe. However, there is considerable evidence from blood meal and host-baited studies that ecoform pipiens can also take blood meals from humans and other mammals. Conversely, ecoform molestus also feeds to a considerable extent on birds, in many cases to the same degree as the pipiens ecoform. Therefore, it may be necessary to take a broader view and consider the potential for both ecoforms to act as enzootic and bridge vectors of medically important arboviruses.

## Figures and Tables

**Table 1:** Mosquito-borne viruses of medical and veterinary importance circulating in Europe, after [23,24,39,40,41,42,43]. Involvement of *Culex pipiens* is highlighted using bold typeface.

Virus	Primary Vertebrate Hosts	Principal Vectors	Medical/Veterinary Importance
Batai virus (Bunyaviridae)	Pigs, horses, ruminants, and isolations from wild birds.	*Anopheles maculipennis* s.l.*, Anopheles claviger* (Meigen, 1804)*, Coquillettidia richiardii* (Ficalbi, 1889)	Mild illness in sheep/goats. Influenza-like illness in humans.
Chikungunya virus (Togaviridae)	Humans as primary reservoirs during epidemics. Non-human reservoirs include monkeys, rodents and birds.	*Aedes aegypti, Aedes albopictus*	Fever, joint pain (also chronic), occasional neurological involvement with some deaths reported.
Dengue virus (Flaviviridae)	Humans.	*Aedes aegypti, Aedes albopictus*	Serotype 1 recorded from Europe. Cases range from asymptomatic to severe haemorrhagic fever.
Inkoo virus (Bunyaviridae)	Mountain hares.	*Aedes communis* (De Geer, 1776)	Influenza-like illness in humans.
Lednice virus (Bunyaviridae)	Birds, primarily of the order Anseriformes.	*Culex modestus* (Ficalbi, 1889)	Unknown, avian fatalities not recorded.
Sindbis/Sindbis-like viruses (Togaviridae)	Birds (Passeriformes), occasionally rodents and amphibians.	*Culex pipiens, Culex torrentium* (Martini, 1925), *Culiseta morsitans* (Theobald, 1901), *Coquillettidia richiardii, Aedes communis, Aedes excrucians* (Walker, 1856), *Aedes cinereus* (Meigen, 1818) and *Anopheles hyrcanus* s.l.	Sporadic illness in birds, including mortality in chickens. Fever, malaise and potentially chronic arthritis in humans, no mortality.
Snowshoe hare virus (Bunyaviridae)	Snowshoe hare, voles, lemmings.	*Aedes cinereus, Aedes vexans* (Meigen, 1830)*, Aedes communis, Aedes punctor* (Kirby, 1837), *Aedes cataphylla* (Dyar, 1916), *Culiseta inornata* (Williston, 1893) and *Culiseta impatiens* (Walker, 1848)	Non-fatal encephalitis in horses. Fever and occasional CNS involvement in humans.
Tahyna virus (Bunyaviridae)	Brown hares, hedgehogs, rodents.	*Aedes vexans*	Influenza-like illness in humans with occasional CNS involvement.
Usutu virus (Flaviviridae)	Birds, particularly the Passeriformes.	*Culex* spp. including *Culex pipiens*	Avian mortality recorded in several species. Limited neuroinvasive cases reported from Italy.
West Nile virus (Flaviviridae)	Wild birds. Mammals including horses and humans incidental hosts.	*Culex pipiens, Culex modestus, Coquillettidia richiardii*	Limited avian mortality in Europe, equine febrile illness with ~25% mortality. Severe neurological disease in <1% human infections.

**Table 2:** Comparative summary of the behavioural and physiological traits of *Culex pipiens* ecoforms.

Ecoform	Trait				
	Mating	Egg-Laying Requirements	Blood-Feeding Preference	Habitat Associations	Overwintering
form pipiens	Eurogamous (mating requires open spaces)	Anautogenous (blood meal required for first egg batch)	Primarily birds	Rural and urban, aboveground	Heterodynamic (undergoes diapause)
form molestus	Stenogamous (can mate in confined spaces)	Autogenous (no blood meal required for first egg batch)	Birds and mammals	Principally urban, aboveground and underground	Homodynamic (active throughout the year)

**Table 3:** Common methods used for the species delineation of the *Culex pipiens* complex.

Method	Target	Primer Sequences	Identification Output	References
**Gel electrophoresis**	Electrophoreticpolymorphisms in various genetic targets, often enzymes	n/a	Provides estimates of genetic differentiation between populations in target genes	[40,92]
**Multiplex end-point PCR**	*ace-2*	**FOR** ACEtorr 5′-TGCCTGTGCTACCAGTGATGTT-3′ **FOR** ACEpip (5′-GGAAACAACGACGTATGTACT-3′) REV B1246s (5′-TGGAGCCTCCTCTTCACGG-3′)	*Cx. pipiens* complex: *Cx. pipiens, Cx. quinquefasciatus, Cx. p. pallens, Cx. australicus, Cx. torrentium, Cx. pervigilans, Cx pipiens/Cx. quinquefasciatus* hybrids	[98]
	CQ11	**FOR** CQ11F (5′-GATCCTAGCAAGCGAGAAC-3′) **REV** pipCQ11R (5′-CATGTTGAGCTTCGGTGAA-3′ **REV** molCQ11R (5′-CCCTCCAGTAAGGTATCAAC-3′	*Cx. pipiens* form pipiens and form molestus	[97]
**PCR-DNA sequencing**	COI	**FOR** LCO1490 (5′-GGTCAACAAATCATAAAGATATTGG-3′) **REV** HCO2198 (5′-TAAACTTCAGGGTGACCAAAAAATCA-3′)	Enables universal identification to species level with comparison to sequence database	[104]
	COI	**FOR** TY-J-1460 (5′-TACAATCTATCGCCTAAACTTCAGCC-3′) **REV** UEA10 (5′ -TCCAATGCACTAATCTGCCATATTA-3′)		[105,106,107]
**PCR-RFLP**	COI	**FOR** COIF (5′-TTGAGCTGGA-ATAGTTGGAACTT-3′) **REV** COIR (5′-CCTCCAATTGGATCAAAGAATGA-3′)	*Cx. pipiens* form pipiens and form molestus, *Cx. torrentium*	[91]
	*ace-2*	**FOR** F1457 (5′-GAGGAGATGTGGAATC CCAA-3′) **REV** B1246 (5′-TGGAGCCTCCTCTTCACGG C-3′)	*Cx. pipiens*, *Cx. quinquefasciatus* and their hybrids	[108]
	*Wolbachia pipientis* markers, *ank2*, *pk1*	**ank2 FOR** (5′-CTTCTTCTGTGAGTGTACGT-3′) **ank2 REV** (5′-TCCATATCGATCTACTGCGT-3′) **pk1 FOR** (5′-CCACTACATTGCGCTATAGA-3′) **pk1 REV** (5′-ACAGTAGAACTACACTCCTCCA-3′)	Five groups of *W. pipientis*: *w*Pip-I to *w*Pip-V	[102,109]
**Real-time PCR**	CQ11	**FOR** *Culex pipiens* (5′-GCGGCCAAATATTGAGACTT-3′) **REV** *Culex pipiens* (5′-CGTCCTCAAACATCCA-GACA-3′) **Probes** *Cx. pipiens* all (59-Cy55-GGAACATGTTGAGCTTCGGK-BBQ-1-39 *Cx. pipiens pipiens* form *pipiens* (5′-JOE-GCTTCGGTGAAGGT TTGTGT-BHQ1-3′) *Cx. pipiens pipiens* form *molestus* (5′-Rox-TGAACCCTCC AGTAAGGTATCAACTAC-BHQ2-3′)	Collectively enables separation *Cx. pipiens* and its ecoforms and hybrids, plus *Cx. torrentium*	[110]
	*ace-2*	**FOR** *Cx. torrentium* (5′ -GACACAGGACGACAGAAA-3′) **REV** *Cx. torrentium* (5′-GCCTACGCAACTACTAAA-3′) **Probe** *Cx. torrentium* (5′-FAM-CGAT-GATGCCTGTGCTACCA-3BHQ1-3′)		
	CQ11	**FOR** Cx_pip_F (5′-GCGGCCAAATATTGAGACTTTC-3′) **REV** Cx_pip_R (5′- ACTCGTCCTCAAACATCCAGACATA-3′) **Probes** Cpp_mol_P (5′-FAM-TGAACCCTCCAGTAAGGTA-MGB-3′) Cpp_pip_P1 (5′-VIC-CACA CAAAYCTTCACCGAA-MGB-3′) Cpp_pip_P2 (5′-VIC-ACACAAACCTTCATCGAA-MGB-3′)	Collectively enables separation *Cx. pipiens* and its ecoforms and hybrids, plus *Cx. torrentium*	[100] (modified from Rudolf et al. [110])
	*ace-2*	**FOR** Cx_tor_F (5′-CTTATTAGTATGACACAGGACGACAG AAA-3′) Cx_tor_R (5′-GCATAAACGCCTACGCAACTACTAA-3′) **Probe** Cx_tor_P (5′-FAM-ATGATGCCTGTG CTACCA-MGB-3′)		

**Table 4:** Blood-feeding hosts of *Culex pipiens* in Europe. Some hosts are non-native to Europe owing to collections in, or close to, captive animal parks.

Order	Family	Genus Species	Common Name	Locations	References
**Mammals**					
Mammal, unidentified	-	*-*	-	Russia	[142]
Artiodactyla	Bovidae	*Capra hircus*	Goat	Spain (Canary Islands)	[143]
		*Ovis aries*	Sheep	Portugal, Turkey	[117,144,145]
		*Bos taurus*	Cow	Portugal, Turkey, Italy, Spain, Germany	[144,145,146,147,148,149]
	Cervidae	*Capreolus capreolus*	Roe deer	Germany	[148]
	Suidae	*Sus scrofa*	Wild boar	Italy, Germany, Spain	[146,147,148]
Carnivora	Canidae	*Canis lupus familiaris*	Dog	Spain, Turkey, Italy, Germany, UK	[145,146,147,148,150,151,152,153,154]
	Felidae	*Felis catus*	Domestic cat	Spain, Czech Republic, Switzerland, Italy	[146,150,152,155,156]
		*Felis silvestris*	Wildcat	Spain	[147]
	Herpestidae	*Herpestes ichneumon*	Egyptian mongoose	Spain	[150]
Chiroptera	Vespertilionidae	*Nyctalus noctula*	Common Noctule	Czech Republic	[155]
Eulipotyphla	Erinaceidae	*Erinaceus europaeus*	European hedgehog	Italy	[146]
Lagomorpha	Leporidae	*Oryctolagus cuniculus*	Rabbit	UK, Germany, Spain	[147,148,157,158]
		*Lepus granatensis*	Granada hare	Spain	[147]
Perissodactyla	Equidae	*Equus caballus*	Horse	France, Italy, Spain	[146,147,159]
Primates	Hominidae	*Homo sapiens*	Human	UK, Spain, Portugal, Czech Republic, Switzerland, Turkey, Italy, Russia, Germany	[142,144,145,146,147,148,149,150,152,154,155,156,160,161,162]
Rodentia	Caviidae	*Cavia porcellus*	Guinea pig	Sweden	[163]
	Muridae	*Rattus rattus*	Rat	Spain	[147]
**Reptiles**					
Reptile unidentified	-	-	*-*	Spain, Italy	[161,164]
Anura	Ranidae	*Rana* sp.	Frog	Czech Republic	[155]
	Hylidae	*Hyla arborea*	European tree frog	Czech Republic	[155]
Squamata	Lacertidae	*Podarcis muralis*	Common wall lizard	Italy	[146]
		*Lacerta* sp.	Frog	Italy	[146]
**Birds**					
Bird, unidentified	-	*-*	-	UK, Spain, Switzerland, France, Russia, Portugal, Sweden	[117,142,151,156,159,160,162,163,164,165,166]
Accipitriformes	Accipitridae	*Hieraaetus pennatus*	Booted eagle	Turkey	[145]
		*Buteo buteo*	Buzzard	Turkey	[145]
		*Neophron percnopterus*	Egyptian vulture	Switzerland	[156]
		*Accipiter nisus*	Eurasian sparrowhawk	Switzerland, Italy	[146,156]
		*Circus aeruginosus*	Western marsh harrier	Czech Republic	[155]
Anseriformes	Anatidae	*Cygnus atratus*	Black swan	Spain	[151]
		*Anas* sp.	Duck	Czech Republic	[155]
		*Anas crecca*	Eurasian teal	Spain	[147]
		*Tachyeres pteneres*	Flightless steamerduck	Switzerland	[156]
		*Anas strepera*	Gadwall	Czech Republic	[155]
		*Anser* sp.	Goose	Czech Republic	[155]
		*Anser albifrons*	Greater white-fronted goose	Czech Republic	[155]
		*Anser anser*	Greylag goose	Czech Republic	[155]
		*Anas platyrhynchos*	Mallard	Portugal, Czech Republic, Switzerland, Italy, Germany	[144,146,148,155,156]
		*Cairina moschata*	Muscovy duck	Spain, Portugal, Italy	[144,146,150]
		*Branta sandvicensis*	Nene	Spain	[151]
Charadriiformes	Laridae	*Larus ridibundus*	Black-headed gull	Spain	[147]
		*Larus fuscus*	Lesser black-backed gull	Portugal	[144]
	Burhinidae	*Burhinus oedicnemus*	Eurasian stone-curlew	Spain	[147]
Columbiformes	Columbidae	*Streptopelia decaocto*	Eurasian collared dove	Spain, Switzerland, Turkey, Italy	[145,146,147,150,152,156,161,164,167]
		*Columba livia*	Rock dove	UK, Spain, Italy	[146,147,154,161]
		*Columba oenas*	Stock dove	UK	[168]
		*Columba palumbus*	Wood pigeon	Spain, Italy, UK	[146,152,168]
Falconiformes	Falconidae	*Falco tinnunculus*	Common kestrel	Portugal	[144]
Galliformes	Phasianidae	*Gallus gallus*	Chicken	Spain, Portugal, Switzerland, Italy, Russia, UK	[144,146,147,149,151,152,156,161,162,168]
		*Pavo cristatus*	Common peacock	Switzerland	[156]
		*Phasianus colchicus*	Common pheasant	Czech Republic, Italy	[146,155]
		*Coturnix coturnix*	Common quail	Czech Republic	[155]
		*Alectoris rufa*	Red-legged partridge	Spain	[150]
		*Alectoris rufa*	Red-legged partridge	Spain	[147]
		*Meleagris gallopavo*	Turkey	Portugal, Italy	[144,146]
	Numididae	*Numida meleagris*	Helmeted guineafowl	Italy	[146]
Gruiformes	Gruidae	*Grus* sp.	-	Spain	[151]
		*Grus grus*	Common crane	Spain	[150]
		*Anthropoides virgo*	Demoiselle crane	Switzerland	[156]
	Rallidae	*Rallus aquaticus*	Water rail	Czech Republic	[155]
		*Gallinula chloropus*	Common moorhen	Italy	[146]
Passeriformes	Acrocephalidae	*Acrocephalus scirpaceus*	Eurasian reed warbler	Czech Republic	[155]
		*Hippolais polyglotta*	Melodious warbler	Portugal, Spain	[144,147]
	Alaudidae	*Galerida cristata*	Crested lark	Spain, Turkey, Portugal	[144,145,147,150]
		*Alauda arvensis*	Eurasian skylark	UK	[168]
	Corvidae	*Corvus corone*	Carrion crow	Switzerland	[156]
		*Garrulus glandarius*	Eurasian jay	Turkey	[145]
		*Pica pica*	Eurasian magpie	Czech Republic, Switzerland, Turkey, Italy	[145,146,155,156,161]
		*Cyanopica cooki*	Iberian magpie	Portugal	[144]
		*Cyanocorax chrysops*	Plush-crested jay	Switzerland	[156]
	Emberizidae	*Miliaria calandra*	Corn bunting	Portugal	[144]
		*Emberiza citrinella*	Yellowhammer	Czech Republic, Germany	[148,155]
	Fringillidae	*Serinus canaria*	Atlantic canary	Portugal	[144]
		*Fringilla coelebs*	Common chaffinch	Czech Republic	[155]
		*Carduelis chloris*	European greenfinch	Spain, Italy	[146,151]
		*Serinus serinus*	European serin	Italy	[167]
		*Carduelis chloris*	Greenfinch	Spain	[147]
	Hirundinidae	*Hirundo rustica*	Barn swallow	Czech Republic, UK	[155,168,169]
		*Delichon urbica*	House martin	Portugal, Czech Republic, Italy, Germany	[144,148,155,167]
	Locustellidae	*Bradypterus tacsanowskius*	Chinese bush warbler	Portugal	[144]
	Motacillidae	*Anthus pratensis*	Meadow pipit	Spain, UK	[147,168]
		*Motacilla alba*	Pied wagtail	Czech Republic, Switzerland	[155,156]
		*Motacilla flava*	Yellow wagtail	UK	[168]
	Muscicapidae	*Erithacus rubecula*	European robin	Italy, Germany	[148,167]
	Oriolidae	*Oriolus oriolus*	Eurasian golden oriole	Italy	[146]
	Paridae	*Cyanistes caeruleus*	Blue tit	Portugal, Czech Republic, Switzerland, Germany	[144,148,149,155,156]
		*Parus major*	Great tit	Switzerland, Italy, UK	[146,156,169]
	Passeridae	*Passer montanus*	Eurasian tree sparrow	Italy	[146]
		*Passer domesticus*	House sparrow	Spain, Portugal, Switzerland, Italy, UK, Germany	[144,146,147,148,149,150,151,152,156,161,164,167,168]
	Sturnidae	*Sturnus* sp.	-	Spain	[147]
		*Sturnus vulgaris*	European starling	Spain, Czech Republic, Italy, UK	[146,150,155,161,168]
	Sylviidae	*Sylvia* sp.	-	Spain	[147]
		*Sylvia communis*	Common whitethroat	Portugal, Czech Republic, Germany	[144,148,155]
		*Sylvia atricapilla*	Eurasian blackcap	Czech Republic, Italy	[146,155]
		*Sylvia borin*	Garden warbler	Portugal	[144]
		*Sylvia melanocephala*	Sardinian warbler	Portugal, Spain	[144,147,150]
	Turdidae	*Turdus merula*	Blackbird	Spain, Portugal, Czech Republic, Switzerland, Italy, UK, Germany	[144,146,147,148,149,150,152,155,156,161,164,167,168,169]
		*Turdus philomelos*	Song thrush	Czech Republic, Germany	[148,155]
Pelecaniformes	Ardeidae	*Nycticorax nycticorax*	Black-crowned night heron	Portugal, Italy, Spain	[144,146,147]
		*Bubulcus ibis*	Cattle egret	Spain	[147]
		*Ardea cinerea*	Grey heron	Czech Republic, UK	[155,168]
		*Ixobrychus minutus*	Little bittern	Spain	[147]
		*Ardeola ralloides*	Squacco heron	Spain	[147]
Piciformes	Picidae	*Jynx torquilla*	Eurasian wryneck	Italy	[146]
Psittaciformes	Cacatuidae	*Nymphicus hollandicus*	Cockatiel	Portugal	[144]
	Psittacidae	*Myiopsitta monachus*	Monk parakeet	Spain	[152]
		*Cyanoliseus patagonus*	Patagonian conure	Switzerland	[156]
Sphenisciformes	Spheniscidae	*Spheniscus humboldti*	Humboldt's penguin	Switzerland	[156]
Strigiformes	Tytonidae	*Tyto alba*	Barn owl	UK	[168]
		*Tyto alba guttata*	Dark-breasted barn owl	UK	[168]
	Strigidae	*Athene noctua*	Little owl	Turkey, Italy	[145,146]
		*Asio otus*	Long-eared owl	UK, Portugal, Spain	[147,149,168]
Suliformes	Sulidae	*Morus bassanus*	Northern gannet	Portugal	[144]

## Appendix A

References used for the production of maps showing country-level presence of the following species:

*Culex pipiens* ecoform pipiens/*Culex pipiens* ecoform molestus hybrids (Figure 2a)
  - Austria [114]
  - Belgium [204]
  - France [102]—hybrids reported from a colony strain originally collected in France
  - Germany [110]
  - Greece [119,205]
  - Italy [118,120]
  - Netherlands [100,115,120]
  - Portugal [117,149]
  - Serbia [206]—methodology used for identification unclear.
  - Spain [116,147,207]
  - Sweden [120]
  - United Kingdom [101,103]
*Culex pipiens*/*Culex quinquefasciatus* hybrids (Figure 2b):
  - Greece [102,121]
*Culex torrentium* (Figure 3)
  - Albania [208]
  - Austria [114,209]
  - Belarus [209]
  - Belgium [89,204,210]
  - Czech Republic [89,209]
  - Denmark [89,209]
  - Estonia [209]
  - Finland [89,209,211,212]
  - France [209,213]
  - Germany [85,89,110,209]
  - Hungary [209,214]
  - Italy [209]
  - Lithuania [209,215]
  - Luxembourg [201,216]
  - Moldova [217]
  - Montenegro [209]
  - Netherlands [89]
  - Norway [209]
  - Poland [128,209]
  - Portugal [209,218]
  - Romania [209,219]
  - Serbia [220]
  - Slovakia [209,221]
  - Spain [222,223]
  - Sweden [79,89,209]
  - Switzerland [89,209]
  - Turkey [224]
  - Ukraine [209]
  - United Kingdom [89,209]
